# Evaluation of an integrated HIV and hypertension management model in rural South Africa: a mixed methods approach

**DOI:** 10.1080/16549716.2020.1750216

**Published:** 2020-04-22

**Authors:** Soter Ameh

**Affiliations:** a Department of Community Medicine, Faculty of Medicine, College of Medical Sciences, University of Calabar, Calabar, Nigeria; b Medical Research Council/Wits University Rural Public Health and Health Transitions Research Unit (Agincourt), School of Public Health, Faculty of Health Sciences, University of the Witwatersrand, Johannesburg, South Africa; c Department of Gobal Health and Population, Harvard T. H. Chan School of Public Health, Harvard University, Boston, Massachusetts, USA

**Keywords:** Chronic, non-communicable diseases, integrated chronic disease management model, HIV, primary health care, health outcomes, Avedis Donabedian, quality of care, Agincourt, South Africa

## Abstract

**Background**: A summary of Soter Ameh’s PhD thesis titled, ‘An integrated HIV and hypertension management model in rural South Africa: A mixed methods approach’ is presented here. In responding to the dual high burden of non-communicable diseases (NCDs) and HIV in South Africa, the national government initiated an integrated chronic disease management (ICDM) model in health facilities as a pilot programme. The aim of the ICDM model is to leverage the successes of the innovative HIV treatment programme for NCDs to improve the quality of care and health outcomes of adult patients.

**Objectives**: The specific objectives of this study were to: (1) determine the quality of care provided in the integrated model in 2013, (2) describe patients’ and operational managers’ perceptions of quality of care in the integrated model in 2013, and (3) assess effectiveness of the integrated model in controlling CD4 counts (>350 cells/mm3) and blood pressure (<140/90 mmHg) of patients from 2011 to 2013.

**Methods**: A combination of quantitative and qualitative methods was used to assess and describe the quality of care in the model. Effectiveness of the model in controlling patients’ blood pressure (BP) and CD4 counts was assessed in selected PHC facilities in the Bushbuckridge municipality in Mpumalanga province, South Africa.

**Results**: The findings showed the suboptimal quality of care in five of the eight priority dimensions of care used as leverage for the NCD programme. The ICDM model had a small but significant effect on BP control for hypertension patients receiving treatment.

**Conclusions**: The HIV programme needs to be more extensively leveraged for hypertension treatment to achieve an optimal BP control in the study area. These findings could have policy relevance for low- and middle-income countries currently undertaking proof of concept studies to demonstrate the feasibility of implementing an integrated chronic disease care model.

## Background

Chronic diseases are defined as diseases that require continuous treatment for many years. They now include HIV/AIDS [1] due to the expansion of the Antiretroviral Treatment (ART) roll-out which has increased life expectancy [2,3].

There is a high burden of HIV/AIDS and NCDs in South Africa and this has implications for its health system [4]. Tackling these conditions seems feasible due to the commonalities related to their progression, prevention, and control. The components of the HIV treatment programme that can be leveraged for NCD care include: programme approaches (peer programmes, defaulter tracing activities, use of multidisciplinary teams as well as engagement of communities), tools (registers, charts, forms and medical records) and systems (monitoring and evaluation, improving quality, drug supply chain and procurement, referral and specimens processing). Others are task-shifting and task-sharing, including the use of community health workers and home-based care [2]. In response to the dual high burden of HIV and NCDs, the government of South Africa introduced a pilot-integrated chronic disease management (ICDM) model in 2011 to leverage the innovative HIV treatment programme for NCDs to improve the quality of care and health outcomes of patients.

This doctoral research presents data on quality of care in the ICDM model as well as the effectiveness of the model in controlling CD4 counts and blood pressure of HIV and hypertension patients, respectively. The findings of this research could provide insight to policymakers, implementers of the ICDM programme, service providers and patients on ways to better integrate HIV and hypertension care.

The following are the specific objectives of this study:

1. To determine the quality of care provided in the integrated model in 2013.
2. To describe patients’ and operational managers’ perceptions of quality of care in the integrated model in 2013.
3. To assess effectiveness of the integrated model in controlling CD4 counts (>350 cells/mm3) and blood pressure (<140/90 mmHg) of patients from 2011 to 2013.

### Conceptual framework

**Figure 1.**
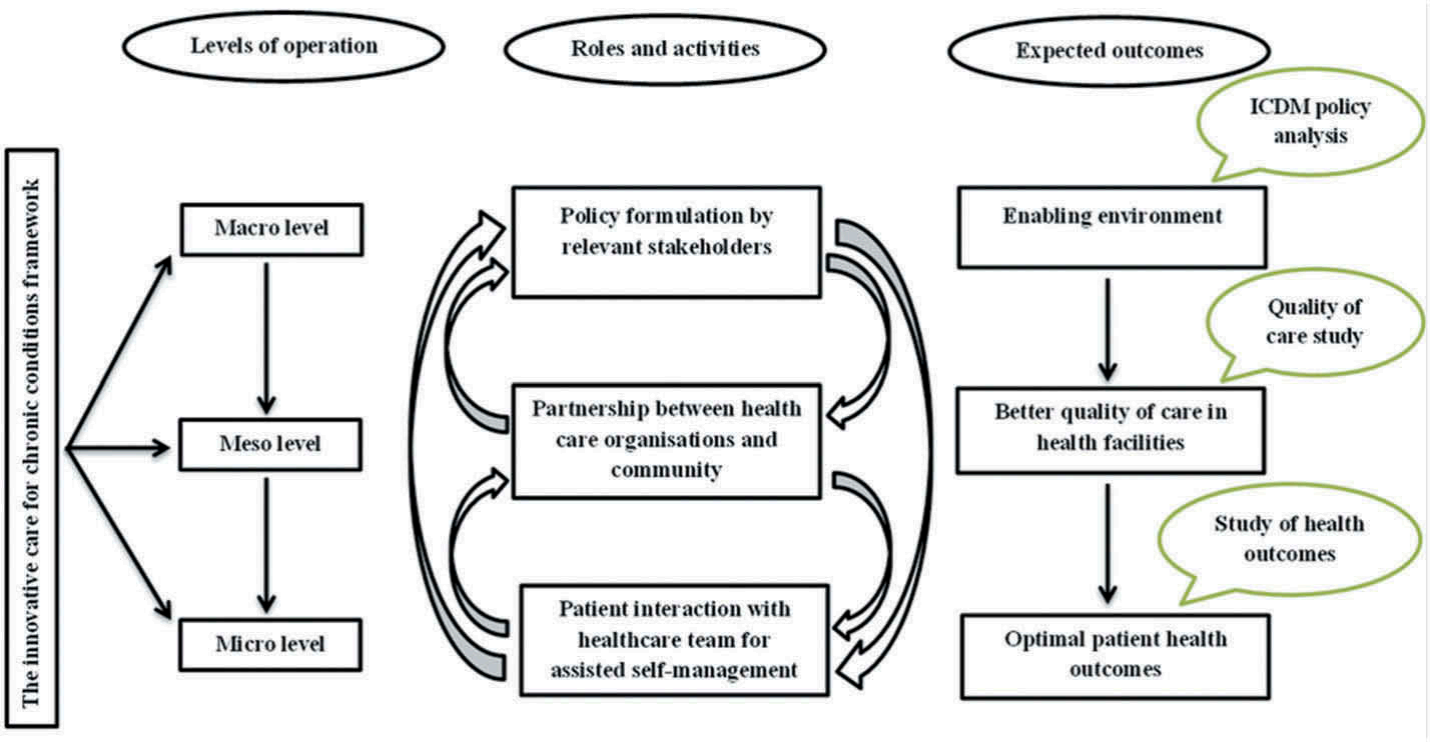
Framework for assessing the integrated model for HIV and non-communicable diseases in South Africa. Adapted from the WHO’s innovative care for chronic conditions (ICCC) framework [1]

This study’s framework (Figure 1) was adapted from the World Health Organization’s Innovative Care for Chronic Conditions (ICCC) framework [1] that underpins the ICDM model which was developed by the National Department of Health (NDoH) in South Africa [5]. The framework highlights the hierarchical levels of interaction between relevant stakeholders, roles and activities of stakeholders, and expected outcomes of these interactions, all of which are necessary for long-term continuity of care for chronic conditions.

The framework shows three levels of health care interactions: patients at the micro-level, health care organisation/community at the meso-level and policy at the macro-level [1]. Optimal outcomes are achieved at the micro-level when there is a partnership between patients and families, health care teams, and community support teams. At the meso-level, resources in communities are brought together for capacity building of health workforce with a view to chronic disease prevention and establishment of a reliable health information tracking system for patients. Governments guide policy-making and set standards for quality of care at the macro-level. These levels of interactions provide opportunities for creating or redesigning a more effective health care system.

The framework also shows how events at different levels influence one another. These interactions are expected to produce a conducive policy environment, better quality of care, and optimal patient health outcomes at the macro-, meso-, and micro-level, respectively.

Two cross-cutting themes have been derived from the three studies in this doctoral research (Table 1):

1. Quality of care in the integrated model: A combination of quantitative and qualitative methods was used to provide a better understanding of quality of care in the integrated model of care.
2. Changes in patients’ health outcomes attributable to the ICDM model: A quantitative study was done to assess the effectiveness of the integrated model of care in controlling patients’ CD4 counts and blood pressure (BP).

**Table 1.** The themes and research objectives in the Vunene study

Themes and research objectives	Papers		
	I	II	III
**Theme 1: Quality of healthcare in the integrated model of care** Research objective 1 (Quantitative method): To evaluate quality of healthcare provided in the integrated model of care in 2013. Research objective 2 (Qualitative method): To assess perception of patients and operational managers regarding quality of healthcare in the integrated model of care in 2013	✓	✓	
**Theme 2: Changes in patients’ health outcomes attributable to the integrated model of care** Research objective 3: To assess the effectiveness of the integrated model of care in controlling patients’ CD4 counts (>350cells/mm3) and blood pressure (<140/90 mmHg) from 2011 to 2013.			✓

### Theoretical framework for evaluating the quality of care in the integrated model

**Figure 2.**
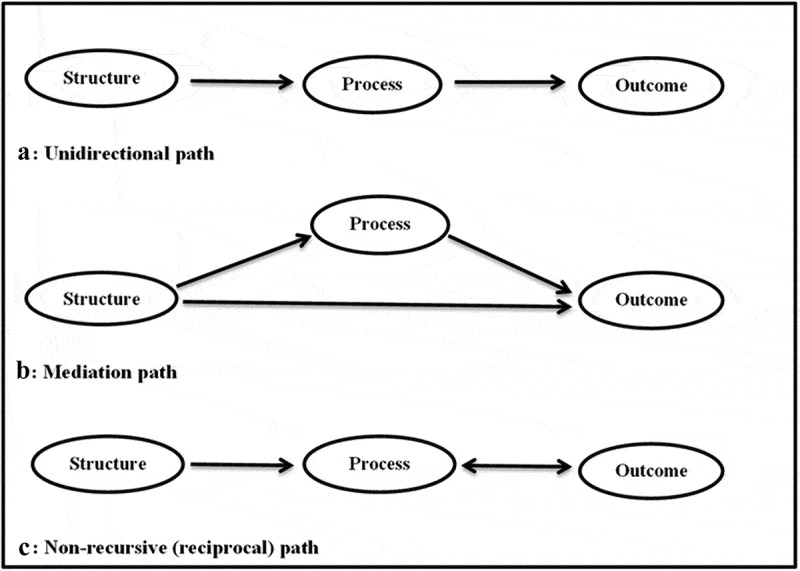
Pathways used to operationalise Avedis Donabedian’s theory of quality of medical care in the intergrated model

Studies 1 and 2 [6,7] used Avedis Donabedian’s relationship between structure, process, outcome (SPO) constructs as the framework [8,9] to draw inferences about the quality of care in the integrated model (Figure 2). The rationale for selecting Avedis Donabedian’s framework is that it is generally the touchstone for evaluating the quality of health care [10], and this was used to operationalise the integrated model in South Africa [5].

## Methods

### The study area and study population

**Figure 3.**
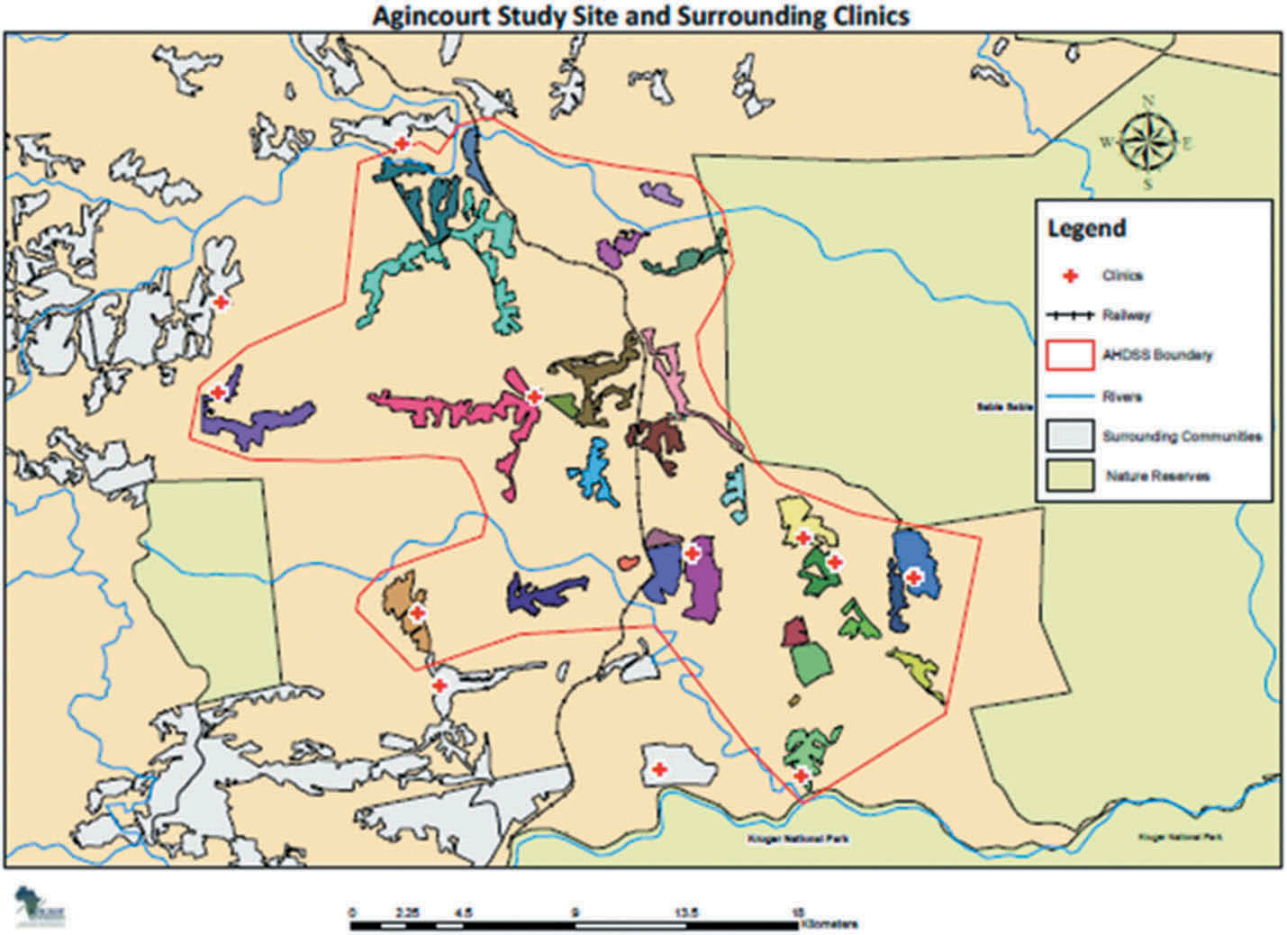
Map of the agincourt health and socio-demographic surveillance system site

The research was conducted in the Bushbuckridge municipality in Mpumalanga province, South Africa. Since 1992, the health and demographic patterns of the population in the Agincourt sub-district in the Bushbuckridge municipality have been monitored by the Agincourt Health and Socio-demographic Surveillance System (HDSS), henceforth referred to as the MRC/Wits-Agincourt Research Unit. The sub-district is 420 km^2^ in area and is situated 500 km northeast of Johannesburg, close to the Mozambican border (Figure 3). About 90,000 people live in 20,000 households in 27 villages in the sub-district [11] where Tsonga is the main language spoken [12,13].

### Data and methods

I designed and implemented this study for my doctoral research. I named it “Vunene” (meaning ‘goodwill’ in Tsonga) because it reflected the goodwill of the Minister of Health in South Africa, Dr. Aaron Motsoaledi who introduced the ICDM model.

### Theme 1: quality of care in the integrated model

Mixed methods (quantitative and qualitative studies) were used to describe the quality of care in the integrated model. This is because mixed methods research can improve understanding of health services by providing a more comprehensive depiction of health services than either method can alone [14]. In this study, both methods were conducted in series with the quantitative quality of care study (exit interviews) preceding the qualitative component. This was to: (1) make it easy to recruit patients for the Focus Group Discussions (FGDs), (2) provide a large number from which prospective FGD participants were to be purposively selected and (3) identify those who overwhelmingly reported satisfaction or dissatisfaction with the quality of care in the integrated care model during the exit interviews for the purpose of further exploring their in-depth perspectives of quality of care using a qualitative method. More specific details of how each method was conducted are shown in the respective sub-sections below.

#### The quantitative component of the quality of care study

A cross-sectional primary study was conducted from August to October 2013. Patients receiving treatment for HIV, hypertension and diabetes in the health facilities 6 months before the initiation of the integrated model in 2011 were identified for recruitment (inclusion criterion). This was to enable assessment of (dis)satisfaction of patients in efforts to gauge changes in the quality of care that can be attributed to the integrated care model. Patients being managed for other chronic diseases, minors less than 18 years of age and the elderly with reduced capacity for comprehension as observed during the informed consent process were excluded from the study.

The study participants also included all the seven operational managers who were professional nurses-in-charge of the health facilities from which patients were recruited. Patients’ satisfaction and operational managers’ satisfaction with the dimensions of care were assessed and patients’ satisfaction scores were used to assess the quality of care in the integrated model using Avedis Donabedian’s quality of medical care framework. The operational managers’ satisfaction scores could not be used to assess quality of care because of their small number (seven).

Of the 17 PHC facilities implementing the integrated model in the municipality, seven facilities serving the communities in the Agincourt sub-district were purposively selected for the study. A sample of 435 was estimated as the minimum sample size after adjusting for 10% non-response [6] using the subjects to variables ratio of 10:1 recommended for studies utilising factor analysis [15,16].

**Figure 4.**
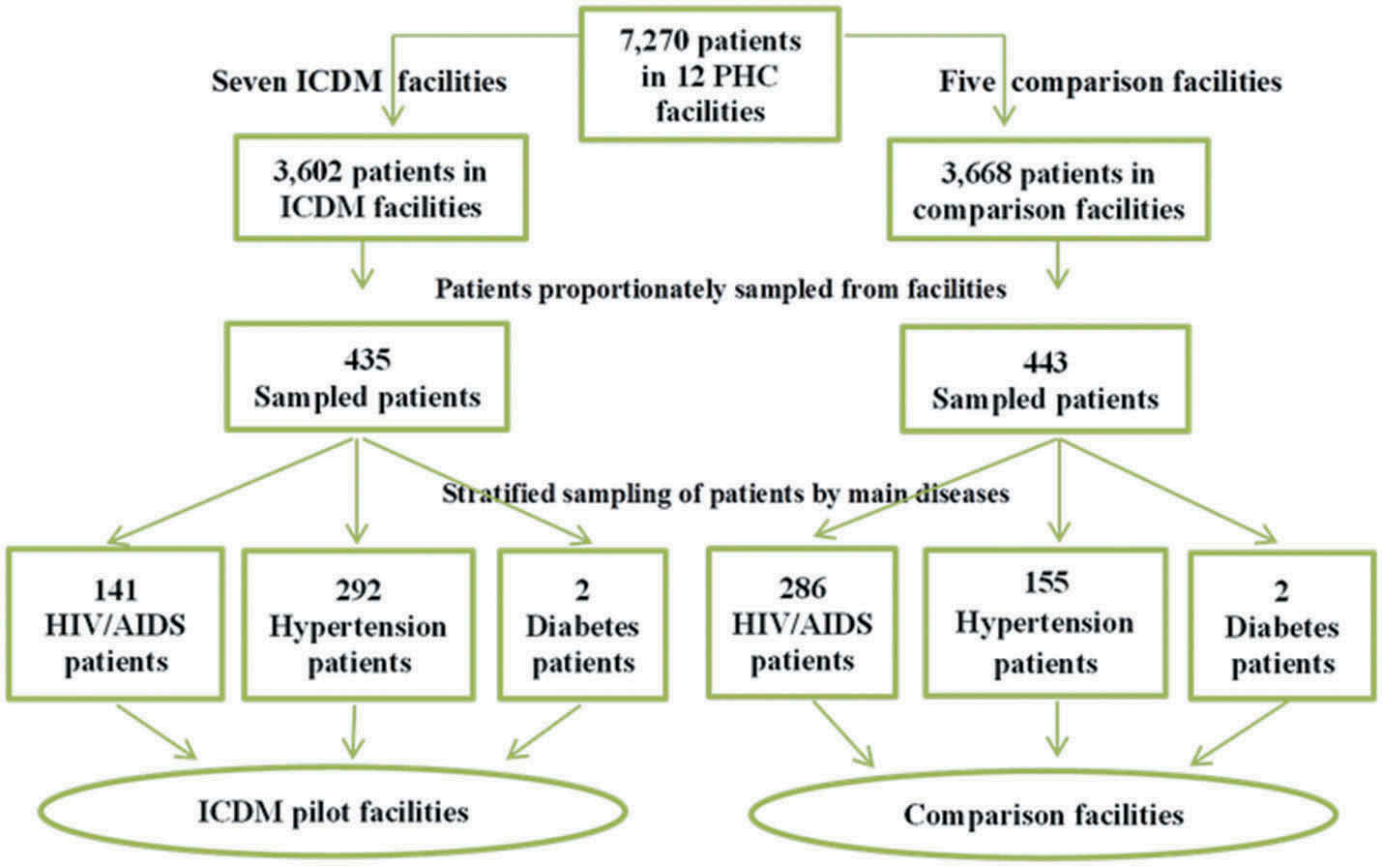
A flow chart of sampling of the study participants in the Vunene study

**Figure 5.**
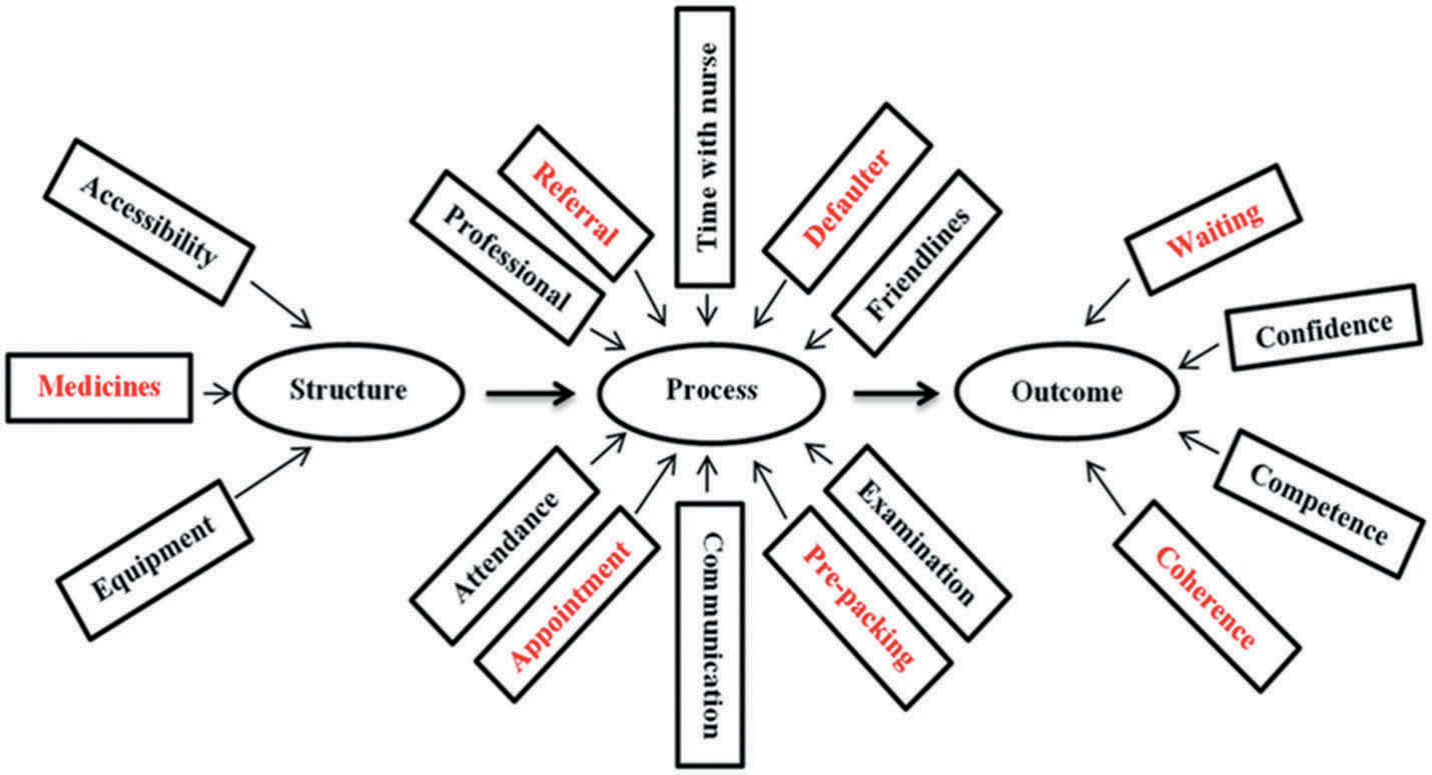
The domains of care in the integrated model assessed under the structure, process and outcome constructs. NB: The domains in red colour indicate the priority areas of the vertical HIV programme leveraged for chronic disease care in the integrated model

Patients were recruited through a multi-stage sampling technique using the July 2013 facility rosters [6], the month before the study was commenced (Figure 4). The 18-item patient satisfaction questionnaire (PSQ-18) developed by Ware et al. [17] was adapted and used for data collection. The adapted study tool succinctly measured satisfaction with 17 dimensions of care for which the SPO constructs were intended (Figure 5).

Eight of these dimensions of care were identified as priority areas. These included structural factors such as critical medicines and equipment; process factors such as referrals, tracing of defaulters; prepacking of medicines and observance of clinic appointments; and outcome factors such as waiting for time and coherence of services [5]. The vertical HIV programme is largely driven by these priority dimensions of care.

Responses to statements during exit interviews were assigned scores based on a five-point Likert scale that ranged from ‘strongly agree’ to ‘strongly disagree’. Satisfaction scores derived from patients’ responses were used to conduct confirmatory factor analysis (CFA) and structural equation modelling (SEM). For positively phrased statements, the respondents were judged to be satisfied if the total relative frequency was 50% or more for ‘strongly agree’ and ‘agree’ responses. The average satisfaction score was 50% on a scale of 0% to 100%.

Satisfaction with the dimensions of care under the SPO constructs were comparatively scored for patients (P) and operational managers (OM). Patients’ satisfaction scores were used to assess the quality of care in the ICDM model through SEM of the relationships between the SPO constructs.

Statistical analysis was done at 5% significance level using Stata 12.0 (College Station, TX, USA). Confirmatory factor analysis and SEM were used to fit the specified path models for the purpose of determining quality of care in the integrated model.

#### The qualitative component of the quality of care study

The qualitative research was a case study of the implementation of the ICDM in pilot facilities based on in-depth perspectives of service users and providers. Capitalising on group interactions, Focused Group Discussions (FGDs) were held for purposively selected participants of similar age to enable in-depth exploration of their lived experiences with service providers on quality of the integrated care model [18]. Seventy (70) of the 435 patients who responded to the quantitative exit interviews in the pilot facilities were selected for seven FGDs with approximately 10 men and women per FGD per health facility. Ten (10) patients were selected for one FGD for clinic defaulters (those who missed three consecutive clinic appointments through the review of clinical records) from the seven clinics. Having being selected after the exit interviews which were held during official working hours (8.00 am – 4.30 pm local time) from Monday to Friday, all 80 patients volunteered to participate in the upcoming FGDs and were briefed about the purpose and scheduled dates of the discussions. It was not possible to conduct FGDs for all the facility managers at a convenient time and venue because of their busy work schedules. Therefore, In-Depth Interviews (IDIs) were held for the operational managers to get their perspectives on quality of care as service providers and facility managers [18]. The FGDs and IDIs were held concurrently in November and December 2013.

The 17 dimensions of care in the adapted PSQ study tool were used as the interview guide in the qualitative component of the quality of care study. Fifty-six (56) of the 70 purposively selected patients participated in the seven FGDs (80% response rate) while five of the 10 selected defaulters participated in the one FGD (50% response rate). Hence, a total of eight FGDs was conducted for 61 of the 80 invited participants (76% response rate). The FGDs were held for 5–9 men and women of similar age with each session lasting 60–90 min. These FGDs were held on Saturdays in places that were centrally located in the health facility catchment areas to enable the patients to freely express and communicate their lived experiences with healthcare services without fear or intimidation [7]. Eight in-depth interviews (each lasting 30 to 40 min) were conducted with the seven female OMs and the health manager of the municipality who was a man [7].

The FGD and IDI transcripts were thematically analysed using MAXQDA 2 qualitative software. A deductive (based on the 17 domains of care in the ICDM model) analytical approach was used for the data analysis. I coded the data and the codes were verified by members of the research team who read and re-read the quotes. The codebook was based on recurring pre-identified themes. Members of the research team verified inconsistent codes through interrogation of the data until an agreement was reached.

### Theme 2: changes in patients’ health outcomes attributable to the ICDM model

Of the 21 PHC facilities where the ICDM model was not being implemented in the Bushbuckridge municipality, five outside the Agincourt HDSS were selected by balloting into the comparison arm. Hence, this sub-study had the ICDM pilot and comparison study arms to enable an objective comparative assessment of patients’ health outcomes (e.g. CD4 counts and BP) which could be attributed to the integrated model of care [19].

The study design was an interrupted time-series (ITS) [20] analysis of data retrieved from facility records in the two study arms to determine the effectiveness of the ICDM model in controlling patients’ CD4 counts and BP.

Overall, 435 and 443 patients were recruited into the ICDM pilot and comparison facilities, respectively (Figure 4). The multi-stage proportionate sampling previously described in the quantitative sub-study on quality of care was applied in the recruitment of the 443 patients in the comparison facilities. A retrospective records review of patients’ CD4 count and BP was performed from January 2011 to June 2013 periods and study numbers generated after unique identifiers of these patients were anonymised.

Eligibility criteria for ART initiation at the time the study was commenced were a CD4 count less than or equal to 350 cells/mm^3^; World Health Organization (WHO) clinical stage 3 or 4; and pregnancy or breastfeeding status [21]. For those on ART, viral load tests were repeated every 12 months and CD4 counts repeated every 6 months for ART monitoring purposes with the expectation that the CD4 counts would be >350 cells/mm^3^. This is referred to as controlled CD4 count in this research.

Hypertension is defined in this study as currently being on antihypertensive medication; or systolic blood pressure ≥140 mmHg or diastolic blood pressure ≥90 mmHg on three separate measurements 2 to 3 days apart [21]. Controlled hypertension is defined as BP <140/90 mmHg.

The main hypothesis was that the ICDM model leads to changes in the CD4 counts and blood pressure of patients receiving care in the pilot facilities with an allowance of a minimum of eight data time points prior to and after the introduction of the ICDM model [22].

The statistical analyses were done using Stata 12.0 at 5% significance level. A controlled segmented regression analysis was used for modelling the data. This method of analysis is used for estimating the effects of longitudinal intervention in interrupted time-series data [20,22]. The purpose of this analysis was to model the monthly average data over time using the autoregressive moving average (ARMA) models to account for autocorrelation inherent in the time-series data [23]. Two time periods were specified: (1) pre-intervention period from January to June 2011 – 6 months before the integrated model was rolled-out, including the month of June 2011 when the model was initiated; and (2) post-intervention period from July 2011 to June 2013–24 months of implementation of the model. Propensity score matching was done to balance the effects of age and sex [24]. Analysis of the data for diabetes patients could not be undertaken due to the small number of patients in both study groups.

## Results

The results of the studies are presented to reflect the thematic areas earlier described.

### Quality of care in the integrated model

The general characteristics of the patients in the ICDM pilot and comparison facilities are shown in Table 2.

**Table 2.** The socio-demographic characteristics of patients in the ICDM pilot and comparison facilities in the Bushbuckridge municipality

Variable	Study groups n (%)			
	ICDM pilot facilities (n = 435)	Comparison facilities (n = 443)	Total (n = 878)	p-value of difference
Age group (years) 18–29 30–39 40–49 50–59 ≥ 60 Missing	19 (4.4) 60 (13.8) 59 (13.6) 84 (19.2) 197 (45.3) 16 (3.7)	39 (8.8) 119 (26.9) 92 (20.8) 85 (19.2) 105 (23.7) 3 (0.6)	58 (6.6) 179 (20.4) 151 (17.2) 169 (19.2) 302 (34.4) 19 (2.2)	<0.001
Gender Female Male	363 (83.4) 72 (16.6)	368 (83.1) 75 (16.9)	731 (83.3) 147 (16.7)	0.881
Education (completed years) No formal education 1–6 > 6 Missing	172 (39.6) 174 (40.0) 71 (16.3) 18 (4.1)	167 (37.7) 169 (38.1) 73 (16.5) 34 (7.7)	339 (38.6) 343 (39.1) 144 (16.4) 52 (5.9)	0.170
Looking for a paid job Yes No Missing	126 (29.0) 291 (66.9) 18 (4.1)	120 (27.0) 301 (68.0) 22 (5.0)	246 (28.0) 592 (67.4) 40 (4.6)	0.725
Type of grant None HIV Disability Old age Missing	202 (46.4) 5 (1.2) 15 (3.5) 195 (44.8) 18 (4.1)	210 (47.4) 8 (1.8) 13 (2.9) 190 (42.9) 22 (5.0)	412 (46.9) 13 (1.5) 28 (3.1) 385 (43.9) 40 (4.6)	0.927
Chronic disease status Hypertension HIV Diabetes Co-morbidities	210 (48.3) 141 (32.4) 2 (0.5) 82 (18.8)	91 (20.5) 282 (63.7) 2 (0.5) 68 (15.3)	301 (34.3) 423 (48.2) 4 (0.5) 150 (17.0)	<0.001

**^†^**Chi-square test p-value of difference between ICDM pilot and comparison facilities

^a^Analysis for diabetes patients was not done because of the small sample size (two in each study arm)

^b^Five patients in the ICDM model facilities were transferred to other facilities also implementing the ICDM model. This was also the case for three patients in the comparison facilities.

^c^Two patients in the ICDM model facilities and one in the comparison arm were transferred to health facilities in other provinces

^d^One HIV patient died in the ICDM model study arm while three deaths (one hypertension and two HIV/AIDS patients) were recorded in the comparison facilities

#### Satisfaction with the structure dimensions of care in the integrated model

**Figure 6.**
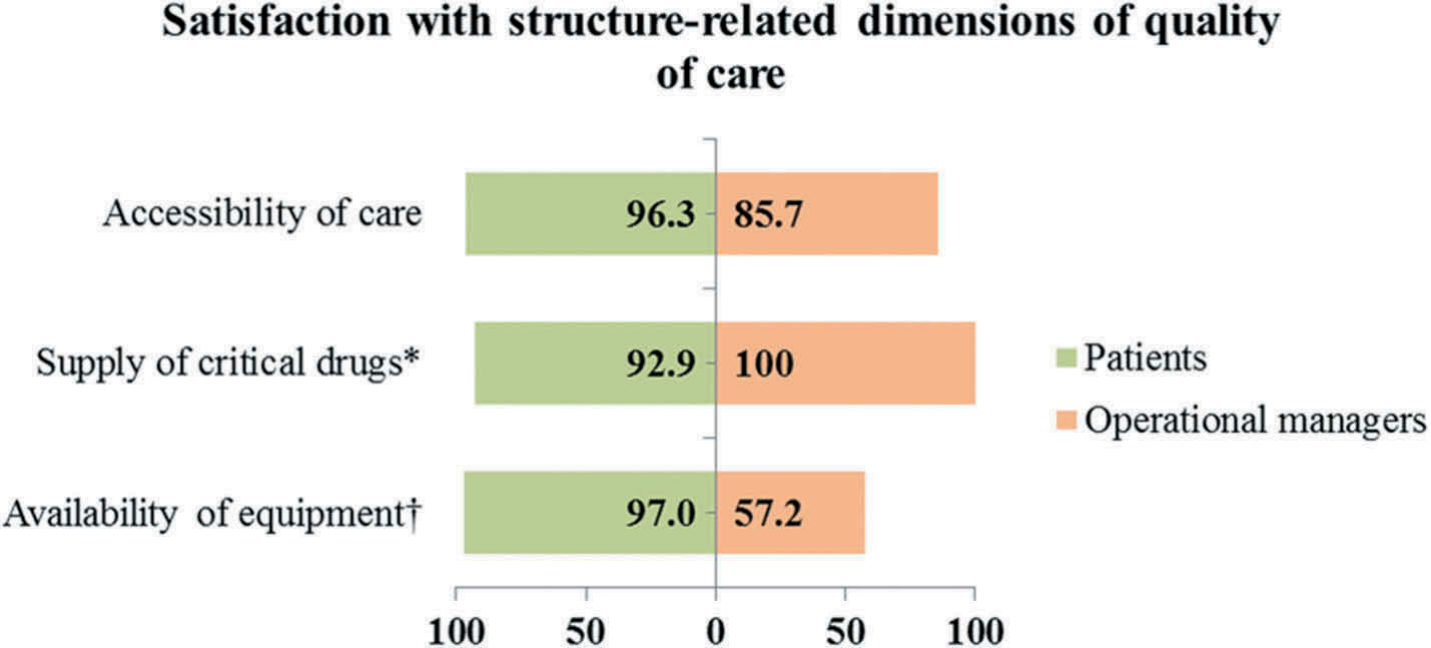
Satisfaction scores of service users and providers with structural domains of care in the integrated model

Patients and operational managers reported satisfaction (scores ≥ 50%) with all the structure-related dimensions of care in the integrated model (Figure 6). A qualitative inquiry on satisfaction with structural dimensions of care showed that users and providers reported occasional antihypertension drug stock-outs and unavailability of or malfunctioning BP apparatus in some health facilities.

When my treatment is not available at the clinic they do tell me that this month my treatment is not available; then they gave me the one that is available that day. When the treatment is not out of stock, they do give me all the treatment that I am getting every month [FGD 1, man].

We have stayed for two to three months without BP machine. They were just giving us treatment without knowing whether our BP was high or not …. It gives us problem when we have to travel to another clinic to check our BP [FGD 7, woman].

[She laughs] what can I say? I think three weeks back Mr. X [a project site manager at institution Y] was here to give us different kinds of BP cuffs because we didn’t have them. I really can’t say that the clinic has all the different medical equipment to take care of all those patients or bring quality nursing care to the patients [IDI 3, woman].

* Priority domains of care in the integrated model.† Statistically significant differences in the satisfaction scores of service users and providers

Staff shortage

Although staff shortage was not contained in the interview guide, it was identified by both service users and providers as a key challenge in delivering quality care in health facilities. A facility manager described making mistakes because of work overload due to staff shortage. A patient described how service providers suffered work-related exhaustion leading to ‘complicated’ behaviour of nurses which negatively impacted the provision of quality care.

I’m alone and I have to do all the programmes with the staff nurse. I’m to manage the deliveries, antenatal clinics, integrated chronic disease clinic, minor illness, immunization and all those programmes. I can’t! … . Sometimes if I am forced to do the work alone I end up making some stupid mistakes (IDI 3, woman).

Today, they [referring to nurses] are two and they get tired and become complicated [FGD 6, woman].

#### Satisfaction with the process dimensions of care in the integrated model

**Figure 7.**
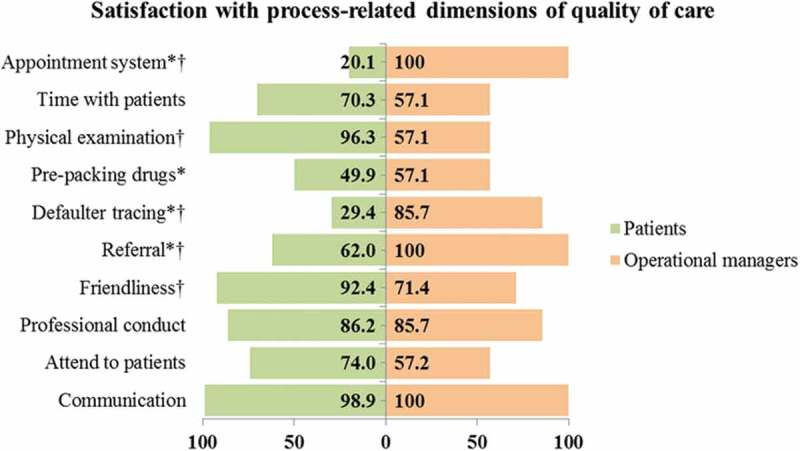
Satisfaction scores of service users and providers with process-related domains of care in the integrated model

Operational managers reported satisfaction with all process-related dimensions of care in the integrated model (Figure 7). However, patients expressed dissatisfaction with defaulter tracing activities (29%) and clinic appointments (20%). The qualitative inquiry of the process aspect of care showed community members stigmatised ill people who were visited by home-based carers (HBCs). Community members associated home visits by HBCs with HIV/AIDS. Therefore, patients responded by not allowing HBCs to visit their homes.

I told them [HBCs] not to come to my house any more. When I tell them something, I expect them to report it to their seniors and not to tell the whole community. So when I’m sick, I will go to the clinic [FGD 2, woman].

* Priority domains of care in the integrated model.† Statistically significant differences in the satisfaction scores of service users and providers

Patients reported that a rigid appointment system made them unable to access services for other illnesses occurring before routine appointment dates. Furthermore, patients who missed their clinic appointments were the last to be attended to in the subsequent visit as nurses would first attend to patients originally scheduled for that day. This punitive measure for clinic defaulters, which was instituted by nurses, was associated with long waiting time during defaulters’ subsequent visit.

When your date is still far you can’t go to the clinic even when you have other illnesses [FGD 3, woman].

When they [nurses] shout at us it is because … they tell you to come today at nine, you find that you miss your appointment date and come at another day. When I missed my appointment and went there the other day, they [nurses] delayed me even when I arrived at the clinic early. All the patients that came after my arrival collected their treatment and went home and left me at the clinic [FGD 1, woman].

A patient reported how a nurse’s unprofessional conduct influenced her perception of the process-related quality of care. In the quote below, a nurse was observed to send patients or cleaners on errands to fetch patients’ medicines from where they are kept, a practice that could lead to swapping of patients’ medication and possible drug toxicity.

Eish! [A popular exclamation in South Africa often used to describe a frustrating or appalling experience] there is a new nurse that arrived at the clinic. She is fat and tall [Man 1 and 2 nod in agreement]. When you are in the consulting room with her [referring to the new nurse], she will send you to go and take the tablets in the locker [referring to where drugs are kept]. Do I know the tablets I have to use? Sometimes she will send a cleaner to go and take the tablets; does the cleaner know the treatment? I have seen it several times and am saying that these nurses are going to kill us [FGD 4, man].

### Satisfaction with the outcome dimensions of care in the integrated model

**Figure 8.**
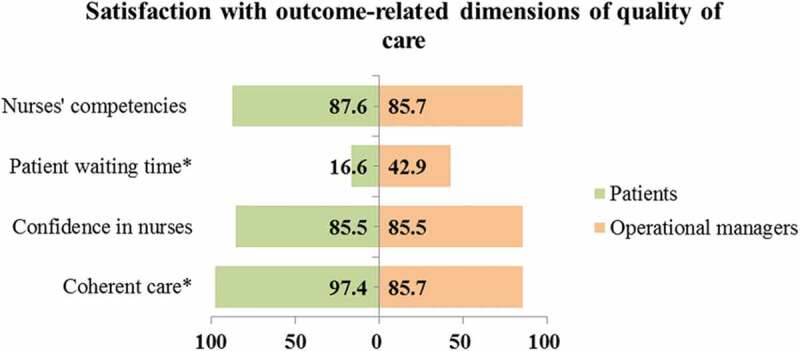
Satisfaction scores of service users and providers with outcome-related domains of care in the integrated model

Patients (17%) and operational managers (43%) expressed dissatisfaction with patient waiting time (Figure 8). In the qualitative analysis, reduced stigma attributable to coherent integrated services and long waiting time were reported as outcome dimensions of care in the integrated model. A facility manager, whose views were similar to other managers, reported that integration of HIV and NCD services was associated with HIV stigma reduction due to the non-segregation of patients in the health facilities.

Patients living with HIV/AIDS are satisfied because they are mixed with those who are having hypertension and diabetes (IDI 6, woman).

* Priority domains of care in the integrated model.

Patients and operational managers observed long patient waiting time in the clinics and attributed this to different factors. From the perspective of patients, long waiting time was attributed to late arrival of filing clerks and nurses to the facilities in the morning; long morning prayer sessions and staff meetings before the commencement of clinical duties; prolonged tea or lunch breaks; nurses’ friends or relatives skipping the queues; and engagement of nurses in buying and selling of household products in the consultation room during consultation hours.

We arrive at six in the morning and stay outside the gate and they will open the gate at eight o’clock. Sometimes they will start to check you at one o’clock. You will get your treatment very late despite early arrival at the clinic [FGD 7, man].

On the other hand, Operational Managers attributed long waiting time to staff shortage and patients missing their previous clinic appointments.

We are booking a certain number of patients and if that number becomes extra because of those who didn’t come on their appointment dates, you find that we have a lot of patients and they [who missed previous appointments] have to wait (IDI 1, woman).

**Figure 9.**
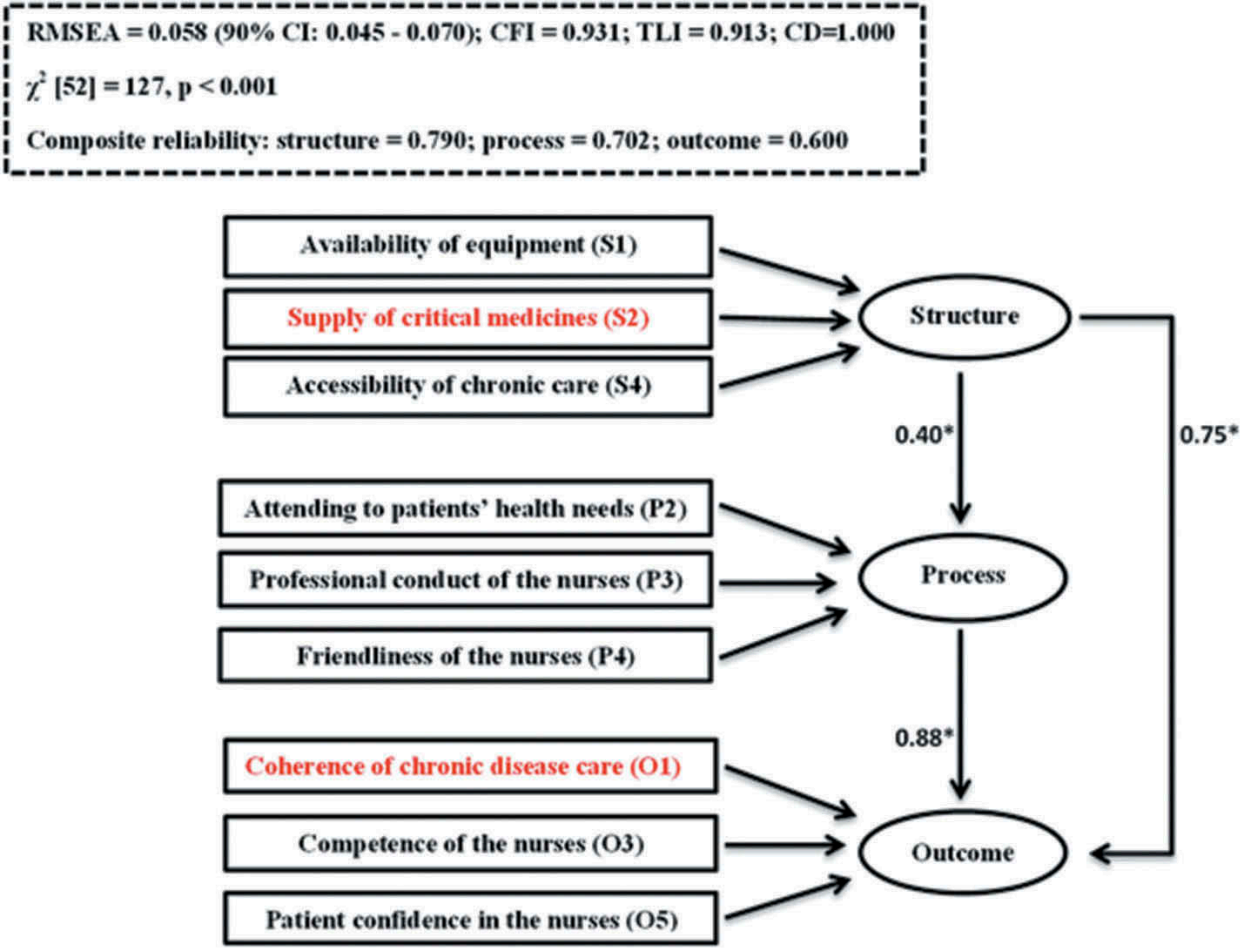
Assessment of correlation between structure, process and outcome constructs

Figure 9 shows how structure, process and outcome constructs relate to one another. The Cronbach’s alpha coefficients of reliability of the variables intended for the structure, process and outcome constructs were 0.790, 0.702 and 0.600, respectively. This is an indication that the variables’ reliability ranged from ‘acceptable’ to ‘good’ and, therefore, were a reliable measure of the constructs they were intended for [25]. There was a reasonable fit of the three pathways as shown in the fit indices (Figure 9).

* Relationships between the constructs represented by the Pearson correlation valuesNB: The domains in red colour are the priority areas in the integrated model.RMSEA- Root Meon Square Error of Approximation (≤0.06 is a good fit)CFI – Comparative Fit Index (CFI≥ 0.90 is a good fit)TLI – Tucker-Lewis Index (TLI≥ 0.90 is a good fit)CD – Coefficient of determination (range 0–1. There is a perfect fit of the data with the model if CD = 1)Cronbach’s alphac coefficient of reliability (≥0.6 is acceptable)

Although only the mediation pathway fulfilled most of the criteria, all the specified pathways fit the data when using at least two criteria (Table 3).

**Table 3.** Goodness of fit of the specified pathways used to evaluate the quality of care in the integrated model

Criteria	Specified path models		
	Unidirectional	Mediation	Reciprocal
χ^2^ test p value > 0.05*	P < 0.001	P < 0.001	P < 0.001
RMSEA value ≤ 0.06	0.064 (90% CI – 0.052–0.077)	0.058)✓ (90% CI – 0.045–0.070)✓	0.059)✓ (90% CI – 0.047–0.070))✓
CFI ≥ 0.90	0.915	0.931 ✓	0.919 ✓
TLI ≥ 0.90	0.892	0.913 ✓	0.910 ✓
CD close to 1.00 (perfect fit is preferred if CD value = 1.00)	0.911 ✓	1.00 ✓	0.632
Ranking**	3^rd^	1^st^	2^nd^

✓Show goodness of fit

**The mediation model ranked first because it fulfilled four criteria (RMSEA, CFI, TLI and CD). In addition, it showed a perfect fit based on CD value of 1.00

**The reciprocal model ranked second because it fulfilled three criteria (RMSEA, CFI and TLI)

**The unidirectional model ranked third because it fulfilled two criteria (CFI and CD). However, it did not show a perfect fit based on CD value of 0.911

### Changes in patients’ health outcomes attributable to the integrated model

**Figure 10.**
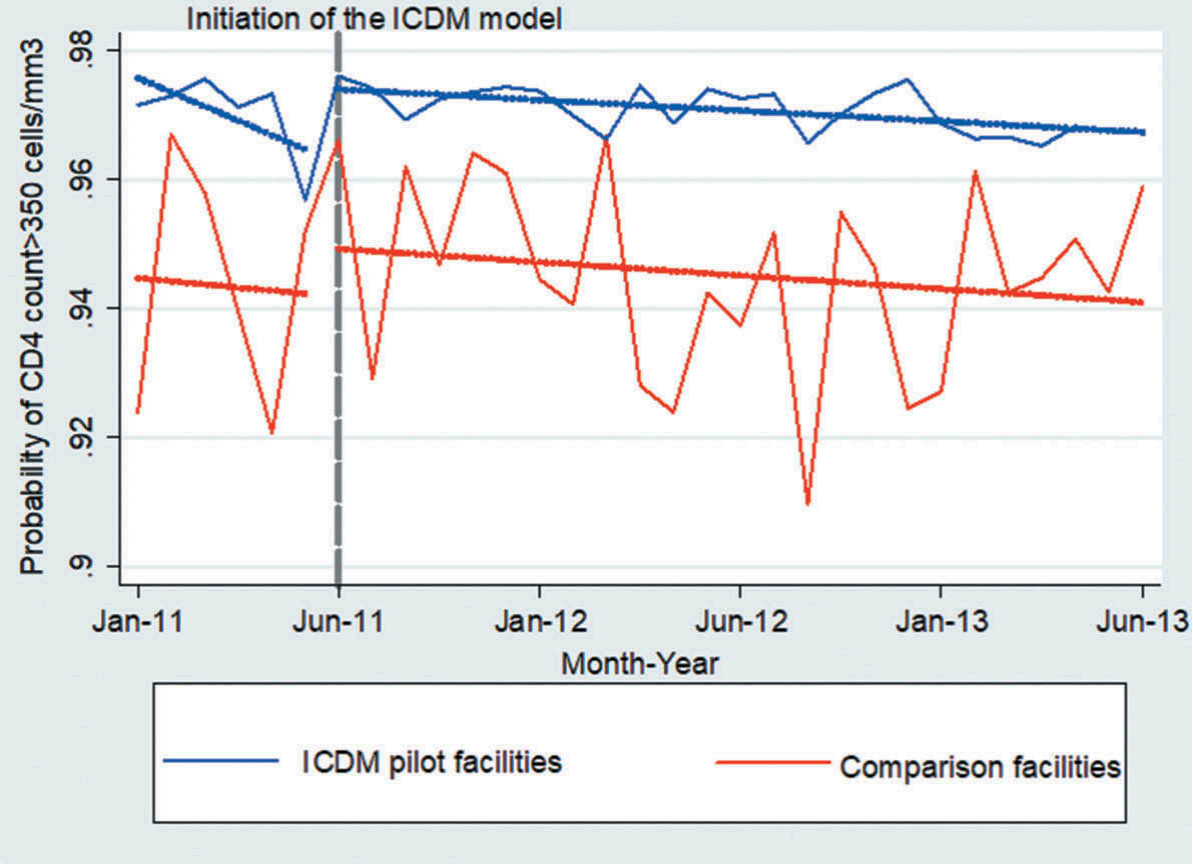
Monthly probabilites of controlling CD4 count (>350 cells/mm^3^) by study groups after propensity score matching

Compared to the comparison facilities (94.5%), the pilot facilities (97.5%) had a higher probability of controlling patients’ CD4 counts at the time the integrated model was initiated and 2 years afterward (94.0% vs. 96.5%) – Figure 10. Table 4 shows that the likelihood of controlling patients’ CD4 counts was 5.7% greater in the pilot than comparison facilities (coef = 0.057; 95% CI: 0.056,0.058; *P* < 0.001). The interaction of study groups and time showed that CD4 count control was greater by 0.2% in the pilot than comparison facilities during the 24 months of implementation of the ICDM model (coef = 0.002; 95% CI: 0.001,0.003; *P* < 0.001).

**Table 4.** The autoregressive moving average model for CD4 count in health facilities in the Bushbuckridge municipality from January 2011 to June 2013

Variables	Coefficient	Standard error	Confidence interval	p-value
Reference attributes Comparison facilities Pre-intervention period				
ICDM pilot facilities	0.057	0.0002	0.056,0.058	<0.001
Post-intervention period	−0.003	0.0001	−0.004,-0.002	<0.001
ICDM pilot*Post-intervention period	0.002	0.0003	0.001,0.003	<0.001
Constant	0.91	0.0001	0.90,0.92	<0.001
Autoregressive moving average (ARMA) modeling				
Autoregressive component (L1)	0.68	0.0212	0.64,0.72	<0.001
Moving average component (L1)	−0.81	0.0185	−0.85,-0.78	<0.001

*Dotted gray line shows the month the integrated model was commenced.

**Figure 11.**
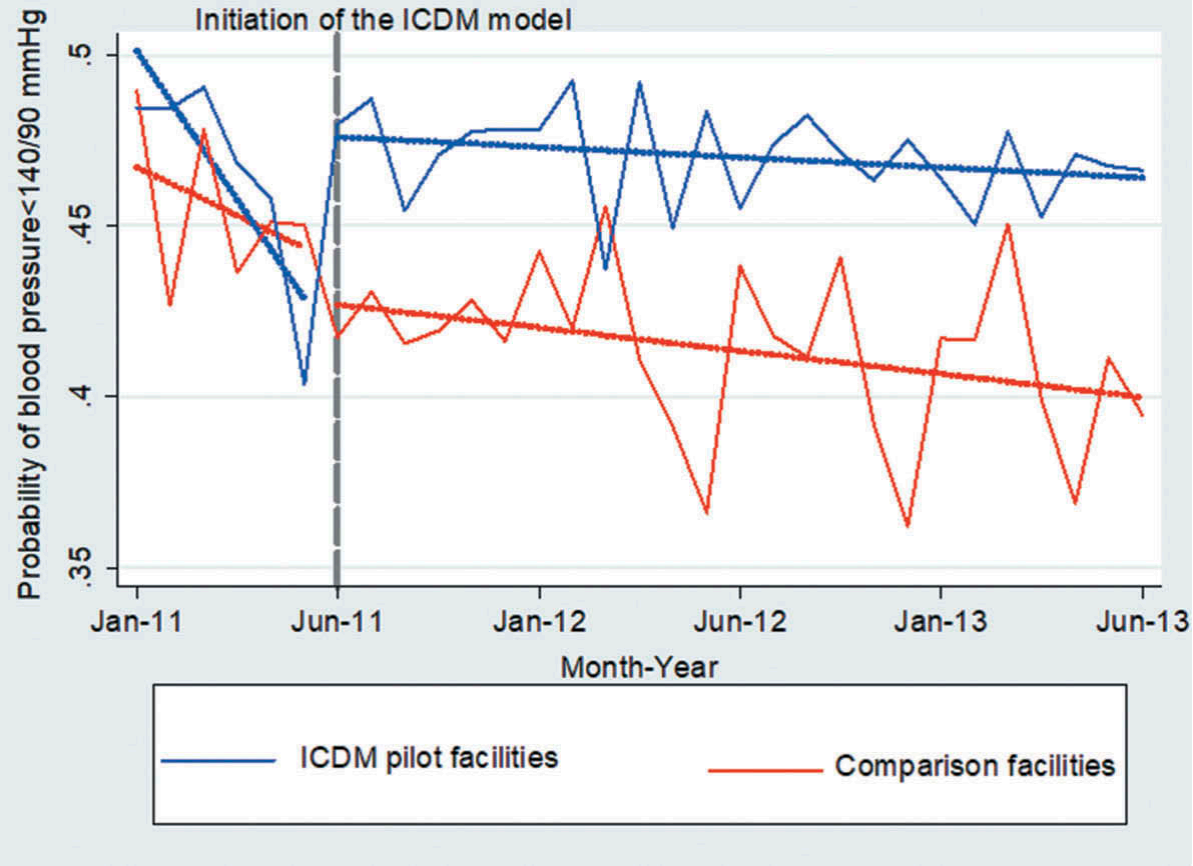
Monthly probabilites of controlling blood pressure (<140/90 mmHg) by study groups after propensity score matching

Comparison of the two study arms showed a consistently higher probability of controlling patients’ BP in the pilot facilities than in the comparison facilities 6 months prior to the commencement of the ICDM model (50% vs. 47%), and 2 years after the model was implemented (47% vs. 40%). The pilot facilities also had less zig-zag fluctuations in BP control than the comparison facilities (Figure 11). In Table 5, the pilot facilities had a 1.0% greater chance of controlling BP than the comparison facilities (coef = 0.010; 95% CI: 0.003,0.016; *P* = 0.002). The interaction between the study groups and time showed the pilot facilities had a 3.6% greater chance of controlling BP than the comparison facilities during the 24 months of implementation of the ICDM model (coef = 0.036; 95% CI: 0.029,0.043; *P* < 0.001).

**Table 5.** The autoregressive moving average model for blood pressure control in health facilities in the Bushbuckridge municipality from January 2011 to June 2013

Variables	Coefficient	Standard error	Confidence interval	p-value
Reference attributes Comparison facilities Pre-intervention period				
ICDM pilot facilities	0.010	0.0031	0.003,0.016	0.002
Post-intervention period	−0.030	0.0030	−0.036,-0.024	<0.001
ICDM pilot*Post-intervention period	0.036	0.0029	0.029,0.043	<0.001
Constant	0.50	0.0030	0.49,0.51	<0.001
Autoregressive moving average (ARMA) modeling				
Autoregressive component (L1)	0.47	0.0576	0.35,0.58	<0.001
Moving average component (L1) Moving average component (L2)	−0.46 0.33	0.0480 0.0272	−0.55,-0.37 0.28,0.38	<0.001 <0.001

*Dotted gray line shows the month the integrated model was commenced.

## Discussion

### Quality of care in the integrated model

The operational managers were satisfied with 16 of the 17 dimensions of care in the quantitative sub-study on quality of care in the integrated model while patients reported satisfaction with 14 dimensions of care. The differences in the satisfaction scores further corroborate evidence-based literature suggesting the need to assess satisfaction with the quality of care from providers and users [26] because of differing views [27].

The use of a qualitative study to triangulate data collection yielded, in part, results that were discrepant from those reported by patients in the quantitative sub-study on quality of care. In the former study, patients reported anti-hypertension drug stock-outs, malfunctioning BP machines, dysfunctional prepacking of drugs, lack of confidentiality in defaulter-tracing activities, unprofessional conduct of nurses, rigid clinic appointment systems and long patient waiting time. This was contrary to the high patient satisfaction scores observed in the facility-based quantitative study. This apparent contradiction may not be due to untrustworthiness of the data and can be explained away (i.e. turns out not to be a contradiction) by the varying locations in which both methods were implemented; hence, the divergence of some of the findings of both methods used [28]. The patients may have had reservations in expressing their negative experiences with health care during the exit interviews in the health facilities for fear of victimisation if service providers overheard them. On the other hand, the FGD participants (all of who took part in the facility exit interviews) may have felt free to communicate their experiences with health services during the community-held FGDs without intimidation.

The only dimension of care in which both users and providers reported low satisfaction scores were patient waiting time. This is supported by a similar study which assessed the quality of service in South African public clinics [29,30]. This suggests that primary health care in South Africa is characterised by long waiting times [31–33], possibly a consequence of operational challenges.

Many African countries have witnessed reductions in HIV-related prejudicial attitudes following ART rollouts [34,35]. However, HIV stigma is still a barrier to HIV treatment in South Africa [36,37]. The HIV-related stigma reported in the communities in this study may be attributed to the defaulter-tracing activities of HBCs, potentially negatively impacting the model. As postulated by Avedis Donabedian, the provider–user interface in this study corroborates the multi-directional relationship between structure, process, and outcome constructs [10].

### Changes in patients’ health outcomes attributable to the integrated model

Reduced HIV stigma in the ICDM facilities may explain the higher percentage in the control of CD4 counts in the pilot than comparison facilities [7]. Reduced HIV/AIDS-related stigma may have led to increased uptake of HIV services because HIV and NCD patients received healthcare in the same consultation rooms as was reported in a Cambodian pilot study [2,38].

There were small but significant differences in the control of CD4 counts and BP in the periods prior to and after the integrated model was commenced. A similar study conducted in Cambodia using cohort analysis showed an increase in median CD4 counts from 53 to 316 cells/mm^3^, and that 68% of hypertension patients on regular treatment had their BP controlled after 2 years of implementation of the pilot study [18]. From a health system perspective, my study does not fully support the Cambodian study because the ICDM model did not show an upward trajectory in the probability of controlling patients’ CD4 counts and BP.

Optimal BP control is difficult to achieve [39–44]. Although there was a very small improvement in BP control, the suboptimal (<50%) control of BP observed in the pilot facilities implies that the purpose of introducing the integrated model is yet to be achieved. The failure to achieve optimal BP control in the study setting may be attributed to health system factors. First, a study showed that the vertical HIV programme was not administratively integrated with the horizontal general health system in South Africa [45]. Second, the quality of care study, which used a quantitative approach, showed that five of the eight priority domains of care in the integrated model were not associated with good quality care in the pilot facilities [6]. Third, facility managers and patients reported that nurses were overburdened by an increased workload resulting from integrated services [7]. Finally, malfunctioning BP machines and antihypertensive drug stock-outs were reported by patients and facility managers in these facilities [7]. Therefore, achieving optimal BP control in the ICDM model will require further strengthening of the broader health system in which the ICDM model is embedded [46].

The decline in the control of CD4 counts and BP before implementation of the model in the pilot facilities was steeper than that in the comparison facilities. This could be due to a ‘crowding-out’ of the quality and quantity of integrated services by routine training activities which typically occur before an interventional programme [46].

### Linkages between study findings and conceptual framework

Patients thought that half of the 17 domains of care in the ICDM pilot facilities were of good quality at the meso-level. An objective assessment of patients’ health outcomes at the micro-level showed a decrease in the downward trajectory in the control of CD4 counts and blood pressure observed in the period before the implementation of the model. Policy analysis at the macro-level designed to examine the role of an ICDM policy in creating an enabling environment towards improved quality of healthcare and patients’ health outcomes was not feasible and needs further research.

### Study limitations

There was incomplete or unavailable facility-level data; and paucity of information on facility-level factors such as comparative data on staffing, patient load, and the inability to obtain at least eight data time points (e.g. 8 months) before the integrated model was commenced. Other limitations were: extrapolations could not be done concerning (dis)satisfaction of professional nurses with services in the integrated model of care because of the small number of facility managers who were interviewed; study findings may not reflect PHC facilities in urban municipalities in Gauteng, Mpumalanga and North West provinces where the pilot model of care was also being implemented; the study sample in the qualitative research was not randomly selected and may not represent patients in the selected health facilities and the qualitative study did not allow the establishment of cause and effect relationships.

### Study strong points

This could be the first study in sub-Saharan Africa to evaluate the quality of care in an integrated model of care and assess the effectiveness of the model in improving CD4 count and blood pressure of patients receiving treatment for HIV and hypertension in PHC facilities. To the best of my knowledge, I am not aware of any studies that have applied Donabedian’s theoretical framework for evaluating an integrated model of care. In my view, the use of both quantitative and qualitative methods to triangulate the data (i.e. collecting data from different population subsets at different time and space) and methodology (i.e. using a combination of quantitative and qualitative methods to gather data) was a major methodological strength of this research. Finally, my thesis contributes to the national and global debates on an integrated health systems approach for the management of chronic diseases.

## Conclusions

Although HIV stigmatisation was reported in the communities due to home visits by home-based carers, the integrated HIV and hypertension treatment model appeared to have had a small but significant effect in controlling the BP of hypertension patients in the health facilities in the study setting. Therefore, there is a need to more extensively leverage the vertical HIV programme to further improve hypertension treatment for an optimal BP control at the facility level.

## Paper context

The HIV vertical programme is not well administratively integrated into the horizontal health system in South Africa; hence, the introduction of the integrated chronic disease management model to scale-up services for NCDs. This doctoral research contributes to ongoing global discourse and could be the first study in SSA to show that an integrated model for HIV and hypertension treatment has a small but significant clinical benefit for hypertension patients. Therefore, the HIV programme should be more extensively leveraged for an optimal hypertension treatment.
